# MicroRNA-targeting in male infertility: Sperm microRNA-19a/b-3p and its spermatogenesis related transcripts content in men with oligoasthenozoospermia

**DOI:** 10.3389/fcell.2022.973849

**Published:** 2022-09-21

**Authors:** Masood Abu-Halima, Lea Simone Becker, Basim M. Ayesh, Eckart Meese

**Affiliations:** ^1^ Institute of Human Genetics, Saarland University, Homburg, Germany; ^2^ Department of Laboratory Medical Sciences, Alaqsa University, Gaza, Palestine

**Keywords:** male subfertility, oligoasthenozoospermia, expression analysis, miR-19a-3p, miR-19b-3p

## Abstract

**Objective:** To elucidate and validate the potential regulatory function of miR-19a/b-3p and its spermatogenesis-related transcripts content in sperm samples collected from men with oligoasthenozoospermia.

**Methods:** Men presenting at an infertility clinic were enrolled. MicroRNA (miRNA) and target genes evaluation were carried out using *in silico* prediction analysis, Reverse transcription-quantitative PCR (RT-qPCR) validation, and Western blot confirmation.

**Results:** The expression levels of miRNA-19a/b-3p were significantly up-regulated and 51 target genes were significantly down-regulated in oligoasthenozoospermic men compared with age-matched normozoospermic men as determined by RT-qPCR. Correlation analysis highlighted that sperm count, motility, and morphology were negatively correlated with miRNA-19a/b-3p and positively correlated with the lower expression level of 51 significantly identified target genes. Furthermore, an inverse correlation between higher expression levels of miRNA-19a/b-3p and lower expression levels of 51 target genes was observed. Consistent with the results of the RT-qPCR, reduced expression levels of STK33 and DNAI1 protein levels were identified in an independent cohort of sperm samples collected from men with oligoasthenozoospermia.

**Conclusion:** Findings suggest that the higher expression of miRNA-19a/b-3p or the lower expression of target genes are associated with oligoasthenozoospermia and male infertility, probably through influencing basic semen parameters. This study lay the groundwork for future studies focused on investigating therapies for male infertility.

## Introduction

About 15% of couples seeking pregnancy are infertile (Sharlip et al., 2002). The defect in about half of them (7%) is associated with the inability of the male partner to produce functional sperms that are capable of fertilization (Krausz and Riera-Escamilla, 2018). The aetiology of male infertility could be attributed to a plethora of congenital and acquired factors (Jungwirth et al., 2018). About 20% of azoospermia and 25% of oligozoospermia cases result from known genetic causes, while the cause is unknown in about a third (known as idiopathic male infertility) (Krausz and Riera-Escamilla, 2018). Alterations in the sequence or expression of single or multiple genes were held responsible; however, their role and precise molecular pathology remain obscure (Zorrilla and Yatsenko, 2013). An intensive effort has been invested to elucidate how those genes are involved in regulating spermatogenesis and sperm function. Recent transcriptome analyses showed that hundreds of genes are predominantly or exclusively expressed in the male germ cells (Djureinovic et al., 2014; Uhlen et al., 2015). These germ cell-specific genes play a crucial role during spermatogenesis and/or sperm function, and their aberrant expression could contribute to male infertility. During spermatogenesis, the gene expression is highly dynamic and strictly regulated via several regulators including microRNAs (miRNAs) (Salas-Huetos et al., 2020). MiRNAs are small non-coding RNAs (∼22 nucleotides in length). These small RNAs are transcribed by RNA Polymerase II from miRNA genes located within protein-coding genes to form a characteristic hairpin structure called primary miRNA. Primary miRNA is processed by Drosha, Dicer, and DGCR8 to form the mature miRNA, which is incorporated into the argonaute protein (AGO) and forms the RNA-induced silencing complex (RISC) (Bartel, 2018; O'Brien et al., 2018). Mature miRNAs regulate gene expression post-transcriptionally through base-pairing with the 3′untranslated region (3′UTR) of messenger RNAs (mRNA) (Bartel, 2018; O'Brien et al., 2018). This interaction results in gene silencing either by destabilization of the mRNA or inhibition of the translation (Bartel, 2018; O'Brien et al., 2018). To date, 2,300 miRNAs have been reported as'' true and/or real'' human mature miRNAs (Alles et al., 2019). These miRNAs are involved in many, if not all, cellular and biological processes, including germ cell development (Bhin et al., 2015), spermatogenesis (Abu-Halima et al., 2013; Abu-Halima et al., 2014a; Abu-Halima et al., 2014b; Abu-Halima et al., 2016; Abu-Halima et al., 2019; Abu-Halima et al., 2020a; Abu-Halima et al., 2021a), early stages of embryonic development (Abu-Halima et al., 2017; Abu-Halima et al., 2020b), women undergoing infertility treatment (Salas-Huetos et al., 2019; Abu-Halima et al., 2021b), and germ cell apoptosis (Reza et al., 2019). Loss of the miRNA processing machinery and/or lack of miRNA biogenesis impair the reproduction process and that loss in function is highly dependent on the stage in which the Dicer and/or Drosha was deleted (Abu-Halima et al., 2021b). Studies reported that the testicular-expressed miRNA changes depending on the stage of spermatogenesis (Hayashi et al., 2008; Maatouk et al., 2008) and that the late meiotic stage and haploid germ cells are the main sources of miRNA production during spermatogenesis (Bouhallier et al., 2010). The haploid mature sperm are stored in the epididymis, whose miRNA expression profile differs from that of the other spermatogenesis stages (Nixon et al., 2015a; Reilly et al., 2016; Reza et al., 2019). Thus, epididymal miRNA might contribute to the maturation and motility of spermatozoa (Reza et al., 2019). Of these miRNAs, hsa-miR-19a-3p and/or hsa-miR-19b-3p (miR-19a/b-3p) were differentially abundant in the sperm, seminal plasma, and testicular tissue of men with different types of unexplained infertility (Wu et al., 2012; Abu-Halima et al., 2013; Abu-Halima et al., 2014a; Abu-Halima et al., 2020b). We have previously suggested that miR-19b-3p might be a potential biomarker for predicting the pregnancy outcome of artificially fertilized embryos (Abu-Halima et al., 2020b). Therefore, the identification of the potential targets of miR-19a/b-3p and the nature of their molecular action will provide insight into the complex downstream effects of miR-19a/b-3p in male fertility/subfertility. It will also allow for identifying potential diagnostic biomarkers and therapeutic targets for male infertility/subfertility. Therefore, in this study, we investigated the differential expression of miR-19a/b-3p and their predicted target genes in men with oligoasthenozoospermia and normozoospermic controls.

## Materials and methods

### Collection and preparation of human sperm samples

A total of 82 men were recruited for the study (mean age ± SD, 25.66 ± 4.21 years; range, 18—35), including 41 oligoasthenozoospermic (subfertile) who attended the IVF lab for infertility treatment and 41 age-matched proven fertile normozoospermic control men. The study complies with the declaration of Helsinki and was approved by the Institutional Review Board (Ha 195/11/updated June 2021) of the Saarland Medical Association. Ethical guidelines were also followed in the conduction of the research. All participants gave their written informed consent before enrolment. Semen samples were obtained from participants by masturbation after 3 days of sexual abstinence, liquefied at 37°C for 30 min, and then processed immediately. All samples were analyzed according to the World Health Organization (WHO) 2010 guidelines for primary semen parameters (liquefaction time, volume, pH, viscosity, agglutination, sperm motility, sperm viability, sperm density, and sperm morphology). These parameters, when taken together, have determined our tested subgroups, i.e., normozoospermia and oligoasthenozoospermia, and none of the included subjects exhibited cells with abnormal morphology (<4%). We excluded samples with a known medical reason for their infertility including Y chromosome microdeletions and other chromosomal abnormalities.

Purification of sperm samples was performed by discontinuous PureSperm^®^ density gradient (Nidacon). Briefly, liquefied semen samples were layered upon a 45:90% density gradient and centrifuged at 500 × *g* for 20 min at room temperature. The recovered pellet was washed twice with Ham–F10 medium supplemented with 5 mg/mL human serum albumin and 0.1 mg/mL penicillin G/streptomycin sulphate (PAN Biotech) and carefully overlaid with 0.75 ml of the same medium. The sample was then kept at 37 °C for 45 min in 5% CO2. One ml of supernatant containing actively motile sperm was then removed and placed in a different tube. After purification of semen samples with density gradient and swim-up, the concentration and progressive motility were evaluated once more. To eliminate possible somatic cells in the ejaculate, semen samples are processed using the somatic cell lysis (SCL) method. This involves incubating the cells in SCL buffer (0.1% sodium dodecyl sulfate and 0.5% Triton X–100 in MilliQ water) on ice for 30 min. A microscopic examination was performed to confirm the absence of residual somatic contaminants with the use of a Makler counting chamber (Irvine Scientific).

### RNA isolation, reverse transcription (RT), and miRNA RT-qPCR

The miRNeasy Mini Kit (Qiagen) was used with a minor modification to extract total RNA including miRNA from sperm. Briefly, 200 µL of the purified sperm samples were homogenized in 700 µL QIAzol^®^ Lysis Reagent (Qiagen) mixed with Dithiothreitol (DTT) (80 mmol/L, Sigma) for 15 min. Subsequently, the procedure was completed according to the manufacturer’s instructions for Qiagen miRNeasy Mini Kit using QIAcube™ Robotic Workstation (Qiagen). DNase I treatment (Qiagen) was performed during the isolation to eliminate any contaminating genomic DNA. Total RNA concentration and purity were determined using Nanodrop-2000 (Thermo Fisher Scientific). Complementary DNA (cDNA) was generated in 20 µL reactions from 75 ng total RNA including miRNA using the *mi*Script Reverse Transcription Kit (Qiagen) according to the manufacturer’s instructions. The 5x *mi*Script HiSpec Buffer was used to generate cDNA from total RNA including miRNA by reverse transcriptase using oligo-dT primers. After RT, the cDNA was diluted with *mi*Script SYBR Green PCR Master Mix (Qiagen) and amplified to detect hsa-miR-19a-3p and hsa-miR-19a-3p. All RT-qPCR experiments were performed using the Liquid Handling Robot QIAgility™ (Qiagen) before performing RT-qPCR using the StepOnePlus™ Real-Time PCR system (Applied Biosystems). Moreover, RT negative controls and no template controls (NTC) were included.

### Prediction of miR-19a/b-3p target genes and network construction

An *in-silico* prediction was carried out to predict the potential target genes of miR-19a-3p (MIMAT0000073) and miR-19b-3p (MIMAT0000074) abbreviated (miR-19a/b-3p), using miRWalk 2.0, which combines 12 target gene prediction algorithms (Dweep and Gretz, 2015). The number of potential target genes of miR-19a/b-3p was shortlisted by including only the target genes predicted by at least 5 different algorithms of the miRWalk. These predicted targets were then checked for the inverse expression levels with miRNA (i.e., miR-19a/b-3p) and mRNA (i.e., inversely correlated expressions of miRNA and mRNA in the RT-qPCR data). As a result, 3,066 potential target genes were predicted. Testis-specific target genes were identified by cross-matching the predicted targets to the Human Protein Atlas (2,237 genes) (www.proteinatlas.org/humanproteome/tissue/testis) and the ToppGene Suite (Chen et al., 2009) algorithms. From this analysis, 130 potential target genes were identified. Out of these 130 potential targets, 82 were selected for RT-qPCR validation based on their functional role in spermatogenesis and/or sperm function, context score of miRNA-mRNA interactions, seed sequence (7-mer sequence or more), and miRNA binding site position within the 3′UTR (left, middle, and right). Using Cytoscape version 3.8.2, the regulatory network was constructed for target genes that have been validated by qPCR and the target genes predicted by at least 5 different algorithms of the miRWalk 2.0.

### Reverse-transcription and pre-Amplification of target genes in the sperm samples

The expression level of multiplex mRNA was quantified by RT-qPCR using the Biomark HD System (Fluidigm Corporation). The Fluidigm^®^ DELTAgene™ Assays (Fluidigm Corporation) was used for the detection of selected target genes according to the manufacturer’s recommendations. Briefly, cDNA was generated in 5 µL reactions from 75 ng total RNA using the Reverse Transcription Master Mix (Fluidigm Corporation) containing both oligo-dTs and random primers, according to the manufacturer’s instructions. Following reverse transcription, 1.5 µL of the generated cDNA was pre-amplified by mixing 1.0 µL of PreAmp Master Mix (Fluidigm Corporation) and 0.5 µL of the Pooled DELTAgene™ Assays mix (500 nM, Fluidigm Corporation) in 5 µL reaction volume. A clean-up step with Exonuclease I (New England Biolabs) was carried out as indicated in Fluidigm’s protocol PN 100–5875 C1. A mixture of 2 µL Exo I containing 0.4 µL Exonuclease I (20 U/μL), 0.2 µL Exonuclease I Reaction Buffer, and 1.4 DNase-free water was added to each 5-μL preamplification reaction using QIAcube™ Robotic Workstation (Qiagen). The preamplification reaction was allowed to digest at 37°C for 30 min before inactivating at 80°C for 15 min using TProfessional Thermocycler (Biometra). Lastly, each sample was diluted with 30.5 µL DNA suspension buffer using the QIAcube™ Robotic Workstation (Qiagen) and was then stored at –20°C for the Biomark™ HD High-throughput RT-qPCR (Fluidigm).

### High-throughput RT-qPCR of target genes in the sperm samples

Following the pre-amplification, RT-qPCR was carried out with 96.96 Dynamic Array™ IFC for Gene Expression (Fluidigm Corporation) as recommended in Fluidigm’s protocol for mRNA (PN 100–9792B1). For each sample, a 6 µL sample mix was prepared, containing 3 µL of 2X SsoFast EvaGreen Supermix with low ROX (Bio-Rad Laboratories), 0.3 µL of 20X DNA Binding Dye (Fluidigm), and 2.7 µL of the pre-amplified sample. A primer stock (100 μM combined forward and reverse primers) was prepared for each assay, and 0.3 µL of the stocks was mixed with 3 µL of 2X Assay Loading Reagent (Fluidigm) and 2.7 µL 1X DNA Suspension Buffer to make assay mixes. Finally, 5 μL of each Assay and Sample Mix were transferred into the appropriate inlets according to the Fluidigm’s recommendation. After loading, the array was placed in the Biomark HD instrument for quantification and detection using a specific thermal cycling protocol for mRNA analysis. GAPDH was used as a reference endogenous control for normalization. The data were then analyzed with Real-Time PCR Analysis Software, Version 4.7.1 (Fluidigm Corporation) as recommended by the provider. A No-Template Control (NTC) and RT-negative control were included in each run.

### Western blot analysis

Western blot analysis was carried out as a small “Proof-of-Concept” to further validate the RT-qPCR using a different set of samples from normozoospermic controls (n = 4) men and oligoasthenozoospermic men (n = 4). Semen samples were thawed on ice and were washed three times with Phosphate-Buffered Saline (PBS). The samples were then centrifuged at 14,000 × *g* at 4°C for 15 min. The pellet was suspended in RIPA buffer (Thermo Fisher Scientific) supplemented with protease inhibitor (Sigma–Aldrich). Then, the samples were sonicated at 20 J for 2 s × 10 at intervals of 10 s and incubated in ice overnight inside the fridge. All samples were then gently mixed for one minute and centrifuged at 14,000 x g at 4°C for 10 min, after which the supernatant was transferred to a new microcentrifuge for Western blot analysis. The concentration of solubilized protein was determined using the bicinchoninic acid assay (BCA assay) (Thermo Fisher Scientific). Twenty-five micrograms of total protein were denatured with Laemmli buffer mixed with β-mercaptoethanol (1:4) (Bio-Rad Laboratories), and the denatured proteins were separated by gel electrophoresis (SDS-PAGE) in a Mini-Protean^®^ TGX Precast Gel (Bio-Rad Laboratories) and subsequently transferred onto polyvinylidene difluoride (PVDF) membranes (Whatman). After that, membranes were blocked for 1 h at RT with TBS Blotto A (Santa Cruz Biotechnology). Membranes were exposed to two primary antibodies namely anti-STK33 antibody [EPR15343] and anti-Dynein intermediate chain 1/DNAI1 antibody [EPR11244-61] diluted in TBS Blotto A overnight at 4°C with agitation. After three washes, each for 15 min, with 1X-Tris buffered Saline with Tween 20 (TBS-T) (Santa Cruz Biotechnology), membranes were exposed to the horseradish peroxidase (HRP)-conjugated secondary antibody (1:3,000, A0545, Sigma-Aldrich) for 1 h. The membranes were cut based on size or stripped and then probed again with GAPDH (14C10) rabbit mAb antibody (1:1,000, 2118S, Cell Signalling Technology), followed by incubation in anti-rabbit secondary antibody. Membranes were then washed three times in 1X-TBS, each for 15 min, developed with enhanced chemiluminescence (ECL) reagent (Cell Signalling Technology), and were exposed to ChemiDoc™ MP Imaging System to detect the chemiluminescence signals (Bio-Rad Laboratories).

### Statistical analysis

GraphPad Prism Software version 9.3.1 (GraphPad Software) was used for statistical analysis. Semen characteristics and expression levels of miRNA and mRNA data were presented as mean ± SD and SE, respectively. To determine the sample size for each group, *a priori* power analysis with α and β error *p* probability of 0.05 was performed, showing that > 30 samples per group are needed for a reliable statistical analysis. An unpaired two-tailed t-test test was used to evaluate the differences in miRNA and mRNA expression levels between subfertile and fertile controls. The relative quantitative method was used to measure the dynamic change of miRNA and mRNA expression levels (Livak and Schmittgen, 2001). RNU6B small nuclear RNA (snRNA) as an endogenous reference miRNA as previously validated for this type of sample (Abu-Halima et al., 2013; Abu-Halima et al., 2014b; Salas-Huetos et al., 2015; Salas-Huetos et al., 2016; Abu-Halima et al., 2019; Abu-Halima et al., 2020a; Abu-Halima et al., 2020b; Abu-Halima et al., 2021a), and only miRNAs with a cycle threshold value (Ct) of < 35 were included in the analysis. Additionally, Spearman correlation analysis was performed to find the association between basic semen parameters and the expression levels of miR-19a/b-3p and significant target genes that showed a lower expression level. Adjustment for multiple testing was performed by controlling the false discovery rate (FDR) according to the approach of Benjamini and Hochberg and a *p-*value of less than 0.05 was considered statistically significant. Finally, the Cytoscape Software (v3.9.1) was used to study the functional enrichment of the DEGs from the dataset.

### Enrichment and network analysis of target genes

The target genes for the miR19a/b-3p were predicted using multiple bioinformatics databases which were comparatively analyzed using the miRWalk algorithm online software (available at: http://mirwalk.uni-hd.de/). Then, we combined the outcomes from the top predicted target genes as predicted by miRWalk and 53 genes which were validated by RT-qPCR to Cytoscape software to find out the interactions among the predicted and validated target genes.

## Results

### Characteristics of the study population

A total of 82 participants (including 41 subfertile men with oligoasthenozoospermia and 41 normozoospermic controls) were recruited for this study. The detailed clinical characteristics of individuals are listed in Table 1. Compared to fertile controls, the subfertile men with oligoasthenozoospermia were significantly different in terms of semen volume (*p* = 0.0047), sperm count (*p* < 0.0001), % motility (*p* < 0.0001), and % sperms exhibiting normal morphology (*p* = 0.0012). Other parameters, such as age and pH were not significantly different.

**TABLE 1:** Semen characteristics of the enrolled men.

Parameters	Normozoospermic controls	Oligoasthenozoospermic men	*p*-value
Age (year)	25.7 ± 4.35	25.6 ± 4.16	0.9871
Volume (mL)	2.32 ± 0.58	1.96 ± 0.35	0.0047
pH	8.15 ± 0.37	8.16 ± 0.31	0.8367
Count (10^6^/mL)	77.3 ± 11.3	9.39 ± 2.41	0.0001
Motility (% motile)	60.5 ± 10.2	20.4 ± 4.3	0.0001
Sperm vitality (eosin) (%)	65.44 ± 5.13	59.90 ± 7.22	0.0002
Morphology (%)	7.29 ± 2.09	5.71 ± 2.21	0.0012

Normozoospermic controls (*n* = 41) and oligoasthenozoospermic men (*n* = 41).

An unpaired two-tailed t-test was used to calculate the *p*-values.

Data were presented as mean ± standard deviation.

*p* < 0.05 was considered statistically significant.

### The expression level of miR-19a/b-3p in the sperm samples of subfertile and fertile men

**FIGURE 1:**
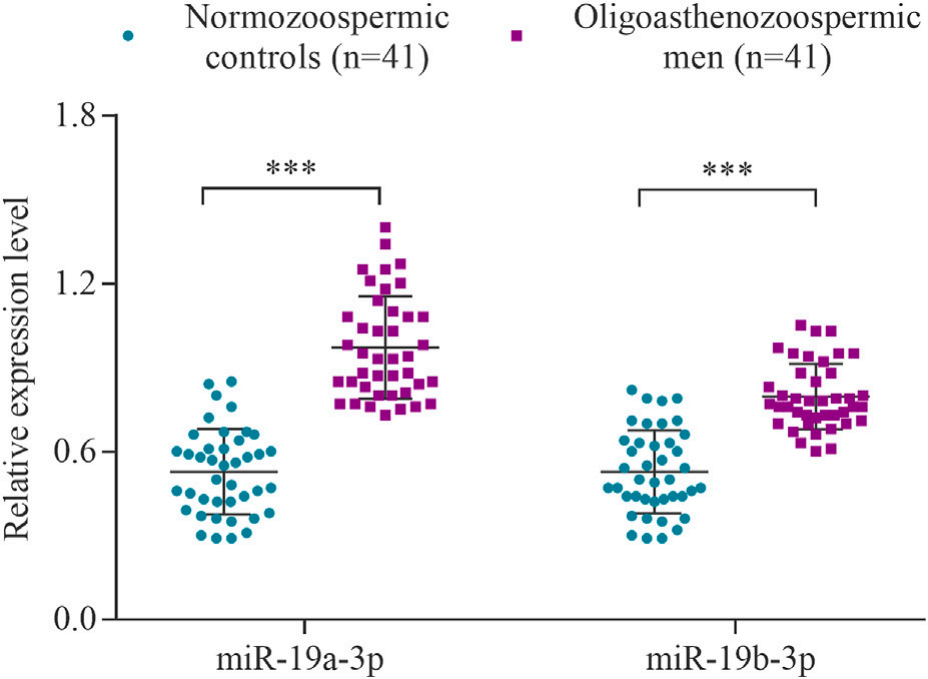
Expression levels of miR-19a/b-3p in the sperm samples collected from men with oligoasthenozoospermia (*n* = 41) and normozoospermia (*n* = 41) as determined by RT-qPCR. Data were presented as the relative expression level 2^−ΔΔCt^ of oligoasthenozoospermic and normozoospermic men. The *p*-value was calculated using an unpaired two-tailed Student’s t-test and *p* < 0.05 was considered statistically significant (***p < 0.001).

The expression levels of the hsa-miR-19a-3p and hsa-miR-19b-3p were determined using RT-qPCR in a cohort of 41 subfertile men with oligoasthenozoospermia and 41 normozoospermic controls. The levels of miR-19a-3p and miR-19b-3p were significantly increased in oligoasthenozoospermic men compared to the normozoospermic controls (*p* = 1.21 × 10^–9^ and 2.49 × 10^–6^, respectively) (Figure 1). The mean expression level was 3.11-fold higher for miR-19a-3p and 2.78-fold higher for miR-19b-3p in subfertile oligoasthenozoospermic men compared to the normozoospermic controls.

### The expression level of target genes in the sperm samples of subfertile and fertile men

RT-qPCR was conducted to confirm the differential expression level of the 82 targets in the same cohort of 41 oligoasthenozoospermic men and 41 normozoospermic controls used for the validation of the higher expression of miR-19a/b-3p. As shown in Supplementary Table S1, out of 82 tested target genes, 53 target genes showed a significant expression level, 19 target genes showed a non-significant expression level, and 10 target genes were omitted from further analysis due to either showing a Ct value of >35 and/or Ct value was not determined. Considering the direction of regulation along with the adjusted significant *p*-value, the differences were significant for 53 target genes (adjusted *p* < 0.05) including 51 targets with lower expression levels and 2 target genes (namely *WNK3* and *MBNL3, p* = 5.15 × 10^–4^ and 1.89 × 10^–3^, respectively) with higher expression levels (Table 2). Moreover, a borderline significant expression level was observed for only one target gene, namely *BRCA2* (*p* = 4.32 × 10^–2^ and adjusted *p*-value = 5.82 × 10^–2^). Using hierarchical clustering with the Euclidean distance measure, we analyzed how the subfertile men and matched fertile controls were related to each other. For this task, we included the significant differential expression target genes (Supplementary Figure S1). We observed two not highly distinct clusters between the subfertile oligoasthenozoospermic men and fertile normozoospermic controls. The first cluster contains lower expression levels detected mostly in fertile controls, and ​the expression levels ranged from lower to higher in the subfertile men. A more detailed distinction between the tested groups based on the clustering dendrogram was, however, not conclusive.

**TABLE 2:** Significantly abundant levels of mRNAs in the sperm samples collected from men with oligoasthenozoospermia compared to men with normozoospermia as determined by RT-qPCR.

Human symbol and name	Fold change	log2	*p*-value	Adjusted *p*-value	Regulation
2A) target genes with lower expression level					
DPYSL5 (dihydropyrimidinase like 5)	0.21	−2.24	1.99 × 10^–6^	1.47 × 10^–4^	Lower
BOD1L2 (biorientation of chromosomes in cell division 1 like 2)	0.20	−2.33	8.07 × 10^–6^	2.03 × 10^–4^	Lower
MED26 (mediator complex subunit 26)	0.31	−1.68	1.36 × 10^–5^	2.03 × 10^–4^	Lower
UBQLNL (ubiquilin-like)	0.15	−2.77	1.37 × 10^–5^	2.03 × 10^–4^	Lower
ATF7IP2 (activating transcription factor 7 interacting protein 2)	0.30	−1.72	2.20 × 10^–5^	2.72 × 10^–4^	Lower
GOLGA6D (golgin A6 family member D)	0.09	−3.43	3.73 × 10^–5^	3.94 × 10^–4^	Lower
SENP8 (SUMO peptidase family member, NEDD8 specific)	0.14	−2.80	5.47 × 10^–5^	4.31 × 10^–4^	Lower
COX8C (cytochrome c oxidase subunit 8C)	0.12	−3.02	5.63 × 10^–5^	4.31 × 10^–4^	Lower
CCDC87 (coiled-coil domain containing 87)	0.12	−3.10	6.22 × 10^–5^	4.31 × 10^–4^	Lower
CSMD1 (CUB and Sushi multiple domains 1)	0.24	−2.06	6.70 × 10^–5^	4.31 × 10^–4^	Lower
RFX4 (regulatory factor X4)	0.28	−1.86	7.31 × 10^–5^	4.31 × 10^–4^	Lower
DCAF12L1 (DDB1 and CUL4 associated factor 12 like 1)	0.11	−3.14	7.58 × 10^–5^	4.31 × 10^–4^	Lower
CCER1 (coiled-coil glutamate-rich protein 1)	0.11	−3.16	1.06 × 10^–4^	5.15 × 10^–4^	Lower
SAMD4A (sterile alpha motif domain containing 4A)	0.27	−1.88	1.17 × 10^–4^	5.15 × 10^–4^	Lower
FAM104A (family with sequence similarity 104 member A)	0.21	−2.27	1.18 × 10^–4^	5.15 × 10^–4^	Lower
TKTL2 (transketolase like 2)	0.12	−3.03	1.31 × 10^–4^	5.15 × 10^–4^	Lower
TMEM215 (transmembrane protein 215)	0.26	−1.93	1.32 × 10^–4^	5.15 × 10^–4^	Lower
HSPA2 (heat shock protein family A (Hsp70) member 2)	0.13	−2.92	1.43 × 10^–4^	5.30 × 10^–4^	Lower
PCDHA7 (protocadherin alpha 7)	0.11	−3.15	2.63 × 10^–4^	8.29 × 10^–4^	Lower
REEP1 (receptor accessory protein 1)	0.11	−3.16	2.69 × 10^–4^	8.29 × 10^–4^	Lower
CSNK1G1 (casein kinase 1 gamma 1)	0.40	−1.33	2.75 × 10^–4^	8.29 × 10^–4^	Lower
POC1A (POC1 centriolar protein A)	0.44	−1.18	2.75 × 10^–4^	8.29 × 10^–4^	Lower
SPATA12 (spermatogenesis associated 12)	0.25	−1.98	2.80 × 10^–4^	8.29 × 10^–4^	Lower
ZNF280B (zinc finger protein 280B)	0.35	−1.52	3.01 × 10^–4^	8.56 × 10^–4^	Lower
AQP5 (aquaporin 5)	0.30	−1.75	4.43 × 10^–4^	1.21 × 10^–3^	Lower
TTLL2 (tubulin tyrosine ligase like 2)	0.30	−1.74	5.06 × 10^–4^	1.30 × 10^–3^	Lower
MYBL1 (MYB proto-oncogene like 1)	0.33	−1.60	5.09 × 10^–4^	1.30 × 10^–3^	Lower
TDRD10 (tudor domain containing 10)	0.35	−1.53	5.96 × 10^–4^	1.39 × 10^–3^	Lower
ELAVL2 (ELAV like RNA binding protein 2)	0.34	−1.56	6.04 × 10^–4^	1.39 × 10^–3^	Lower
ASAP2 (ArfGAP with SH3 domain, ankyrin repeat and PH domain 2)	0.40	−1.33	6.20 × 10^–4^	1.39 × 10^–3^	Lower
PRSS54 (serine protease 54)	0.30	−1.74	6.26 × 10^–4^	1.39 × 10^–3^	Lower
CPEB1 (cytoplasmic polyadenylation element binding protein 1)	0.30	−1.75	6.40 × 10^–4^	1.39 × 10^–3^	Lower
SPIRE1 (spire type actin nucleation factor 1)	0.44	−1.19	1.05 × 10^–3^	2.15 × 10^–3^	Lower
HSF5 (heat shock transcription factor 5)	0.36	−1.47	1.36 × 10^–3^	2.72 × 10^–3^	Lower
USP6 (ubiquitin specific peptidase 6)	0.33	−1.61	2.24 × 10^–3^	4.36 × 10^–3^	Lower
GOLGA6A (golgin A6 family member A)	0.31	−1.69	2.41 × 10^–3^	4.54 × 10^–3^	Lower
GNAT1 (G protein subunit alpha transducin 1)	0.43	−1.23	2.45 × 10^–3^	4.54 × 10^–3^	Lower
UBN2 (ubinuclein 2)	0.53	−0.92	2.93 × 10^–3^	5.17 × 10^–3^	Lower
C2orf42 (chromosome 2 open reading frame 42)	0.50	−1.01	3.22 × 10^–3^	5.54 × 10^–3^	Lower
C22orf31 (chromosome 22 open reading frame 31)	0.34	−1.56	5.00 × 10^–3^	8.41 × 10^–3^	Lower
FHL5 (four and a half LIM domains 5)	0.44	−1.19	5.21 × 10^–3^	8.57 × 10^–3^	Lower
DNAI1 (dynein axonemal intermediate chain 1)	0.41	−1.27	5.50 × 10^–3^	8.85 × 10^–3^	Lower
MCHR2 (melanin-concentrating hormone receptor 2)	0.42	−1.26	1.02 × 10^–2^	1.61 × 10^–2^	Lower
SCML2 (Scm polycomb group protein-like 2)	0.53	−0.90	1.11 × 10^–2^	1.71 × 10^–2^	Lower
DEPDC1 (DEP domain containing 1)	0.58	−0.78	1.16 × 10^–2^	1.75 × 10^–2^	Lower
STK33 (serine/threonine kinase 33)	0.40	−1.31	1.56 × 10^–2^	2.31 × 10^–2^	Lower
DDHD1 (DDHD domain containing 1)	0.54	−0.89	2.74 × 10^–2^	3.97 × 10^–2^	Lower
FAM169A (family with sequence similarity 169 member A)	0.67	−0.58	2.99 × 10^–2^	4.25 × 10^–2^	Lower
FSHR (follicle stimulating hormone receptor)	0.50	-0.99	3.09 × 10^–2^	4.31 × 10^–2^	Lower
ODF4 (outer dense fiber of sperm tails 4)	0.45	−1.16	3.27 × 10^–2^	4.48 × 10^–2^	Lower
BRCA2 (BRCA2 DNA repair associated)	0.55	−0.86	4.32 × 10^–2^	*5.82 × 10* ^*–2*^	Lower
2B) target genes with higher expression level					
WNK3 (WNK lysine deficient protein kinase 3)	1.89	0.92	1.26 × 10^–4^	5.15 × 10^–4^	Higher
MBNL3 (muscleblind like splicing regulator 3)	2.21	1.14	8.96 × 10^–4^	1.89 × 10^–3^	Higher

Normozoospermic controls (*n* = 41) and oligoasthenozoospermic men (*n* = 41).

An unpaired two-tailed t-test was used to calculate the *p*-values.

False Discovery Rate (FDR) correction was used to adjust the *p*-values.

Significant changes in abundance levels are shown with an adjusted *p*-value < 0.05 or a border-line *p*-value (*italic font*).

### Western blot analysis

**FIGURE 2:**
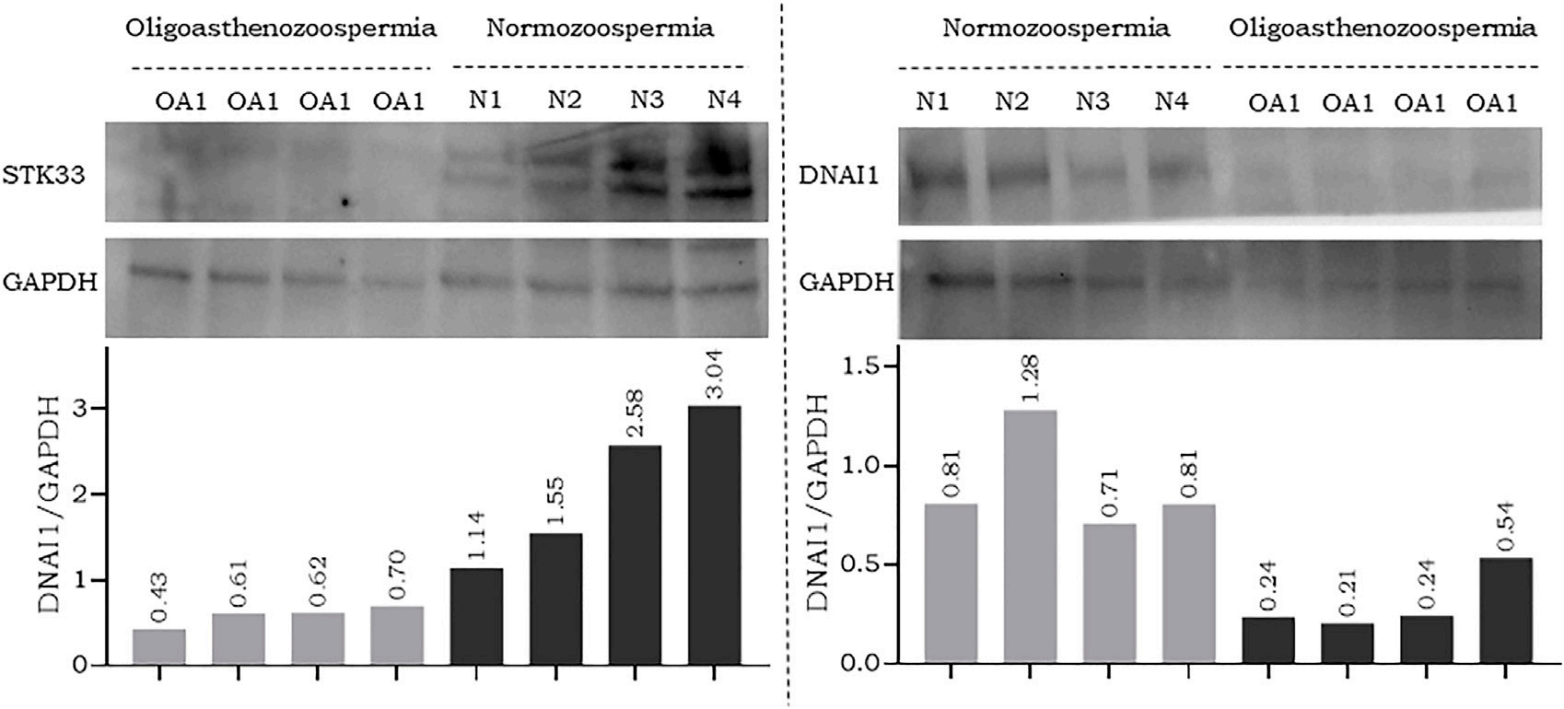
Western blot analysis and quantification of flagellar and cilia assembly proteins STK33 (approximately 58 kDa), DNAI1 (approximately 79 kDa), and the endogenous control GAPDH (approximately 37 kDa) in semen samples collected from men with oligoasthenozoospermia (OA) and normozoospermia (N).

To further confirm the results of RT-qPCR, a new set of semen samples collected from oligoasthenozoospermic men (n = 4) and four normozoospermic controls (n = 4) was analyzed by Western blot. Western blot results showed that the expression levels of anti-STK33 and anti-DNAI1 were consistent with the results of the RT-qPCR analysis. Specifically, a reduction in STK33 and DNAI1 expression levels was observed, as shown in Figure 2 and Supplementary Figure S2. The average quantified protein level of STK33 was 2.15 ± 0.82 in normozoospermic men versus 0.59 ± 0.10 in oligoasthenozoospermic men (*p* = 0.017), and for DNAI1 it was 0.9 ± 0.22 in normozoospermic men versus 0.31 ± 0.13 in oligoasthenozoospermic men (*p* = 0.007) Figure 2.

### Correlation of expression levels (ΔCt) of miRNAs and target genes with basic semen parameters

We next analyze correlations between the expression level of significant genes and the basic semen parameters i.e., sperm count, motility, and morphology. Spearman correlation analysis showed a strong correlation between the miR-19a/b-3p and the sperm count and motility, and a weak correlation with the number of cells exhibiting normal morphology and sperm vitality (eosin, %) (Table 3). Furthermore, a weak to moderate correlation between the significant target genes associated with sperm motility and basic semen parameters. Specifically, correlation analysis showed that the lower the expression level of the 51 genes, the lower the sperm count, motility, and the number of cells exhibiting normal morphology (*p* < 0.0001) (Supplementary Table S2). By contrast, the higher the expression level of the 2 genes namely (*WNK3* and *MBNL3*), the lower the sperm count, motility, and the number of cells exhibiting normal morphology (*p* < 0.0001) (Supplementary Table S2). Similarly, a weak to moderate correlation was observed between the expression level of miR-19a/b-3p and the significant target genes associated with sperm motility (Supplementary Table S3).

**TABLE 3:** Correlation analysis between the expression level of miRNAs and basic semen parameters.

Spearman’s correlation		Count (10^6^/mL)	Motility (% motile)	Sperm vitality (eosin) (%)	Morphology (%)
miR-19a-3p	Correlation coefficient	−0.76	−0.73	−0.45	−0.29
	*p* value	0.0001	0.0001	0.0001	0.0076
miR-19b-3p	Correlation Coefficient	−0.64	−0.66	−0.46	−0.25
	*p* value	0.0001	0.0001	0.0001	0.0249

Spearman correlation analysis.

An Unpaired two-tailed t-test was used to calculate the *p*-value.

A significant change in abundance level was considered with a *p*-value < 0.05.

### Regulatory network construction

**FIGURE 3:**
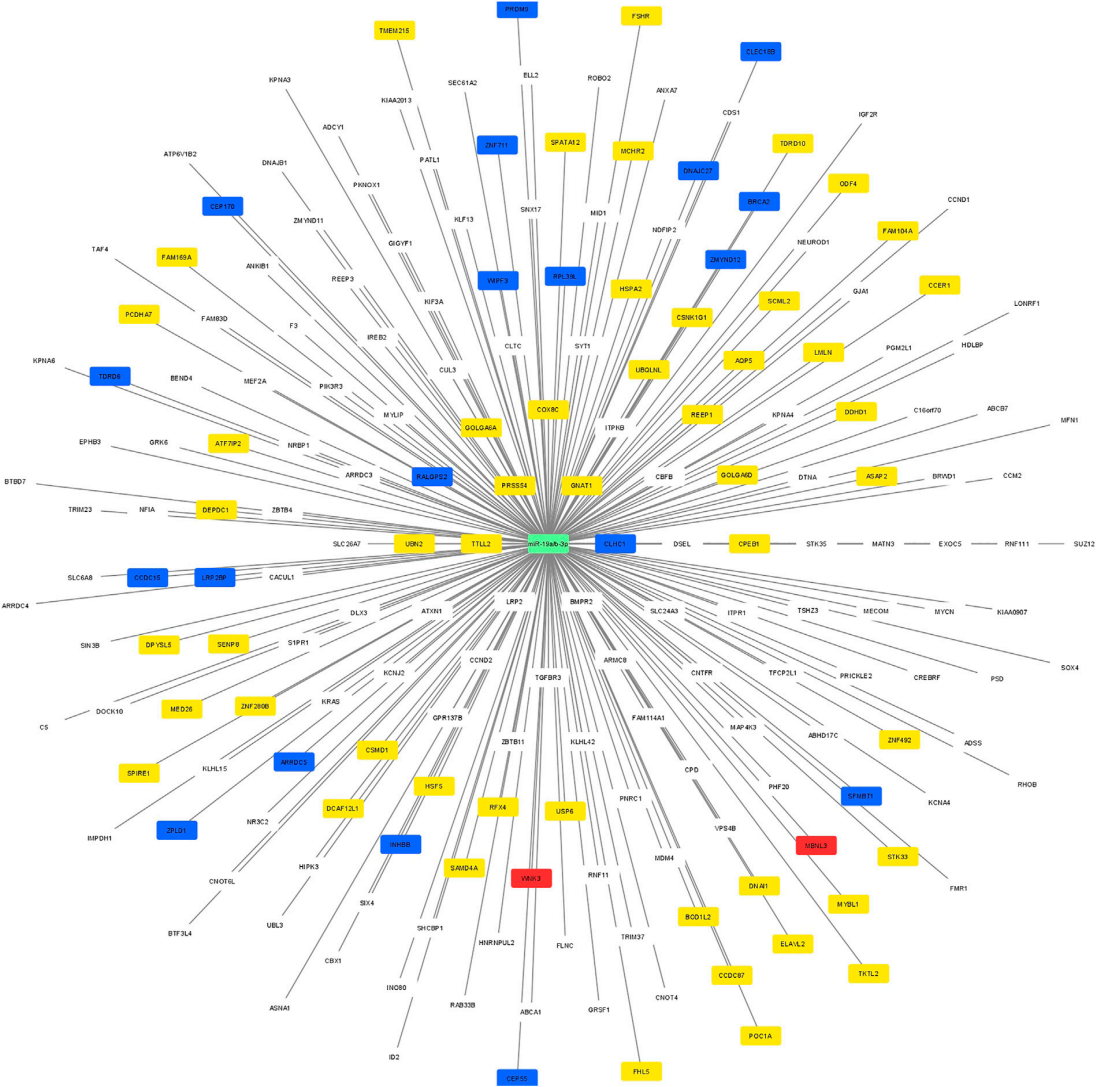
A regulatory network between the potential target predicted genes and the 74 genes which were included in the RT-qPCR. The yellow nodes showed the significantly lower expressed genes, the red nodes showed the significantly higher expressed genes, and the blue nodes represent the non-significant genes, as resulted from RT-qPCR.

Using Cytoscape software, a regulatory network was constructed between the potential target genes which were predicted by at least 5 different algorithms of the miRWalk, and the 53 genes which were validated by RT-qPCR. A clear distinction between the predicted potential target genes and the validated target by RT-qPCR based on the regulatory networking was, however, not possible. Therefore, the regulatory network was constructed based on the highest differential 250 target genes and 2 miRNAs (miR-19a/b-3p). The significant differential expression value (log2 fold change and adjusted *p*-value) for each validated gene is shown in Figure 3. The yellow nodes showed the significantly lower expressed genes, the red nodes showed the significantly higher expressed genes, and the blue nodes represent the non-significant genes, as resulted from RT-qPCR.

## Discussion

Since their discovery, miRNAs have been characterized in several cell types and tissues, and their biomolecular functions are being defined in an expanding number of biological processes (Bartel, 2018). Despite their differential expression in hundreds of species and their role as a crucial regulator during the developmental processes and diseases, the comprehension of their functions in detail needs to be gathered. Update to date, many hundred genes are involved in the regulation of the spermatogenesis process, some of which are not exclusively expressed in the testis and control multiple physiological processes, and many other genes are exclusively expressed (Djureinovic et al., 2014; Uhlen et al., 2015). Aberration of gene expression at any stage of this complex and highly regulated process can lead to male subfertility. Additionally, it is not surprising that miRNAs also play a crucial role in regulating the process of sperm production at different levels, and their aberrant expression can lead to male subfertility. Thus, it is legitimate to hypothesize that differential miRNA patterns can be used to a large extent as promising tools for the treatment and diagnosis of male infertility (Salas-Huetos et al., 2020). Recently, hsa-miR-19a and hsa-miR-19b were highly dysregulated in purified sperm (Abu-Halima et al., 2013), seminal plasma (Wu et al., 2012), and testicular tissue (Abu-Halima et al., 2014a) of men with different types of unexplained infertility in comparison to control fertile men. The miR-19 family members, miR-19a and miR-19b-1 are located within the miR-17–92 gene cluster, which regulates spermatogenesis (Novotny et al., 2007; Tong et al., 2012; Liu et al., 2015; Xie et al., 2016). Specifically, miR-19a-3p was reported as an important regulator of the immune system within the testis (Parker and Palladino, 2017). In agreement with other studies (Cimadomo et al., 2019; Zhou and Dimitriadis, 2020), miR-19b-3p was differentially expressed in the spent culture media after embryo transfer and showed an AUC value for predicting positive pregnancy outcomes, both, from spent culture media and sperm collected from infertile couples attending infertility treatment (Abu-Halima et al., 2020b). These findings lead us to hypothesize that the miR-19 family could play an essential role in reproduction, especially in human spermatogenesis and embryonic development, and could influence male fertility when aberrantly expressed.

In our study, the higher expression level of miR-19a/b-3p was observed in sperm samples collected from oligoasthenozoospermic men compared to normozoospermic men using RT-qPCR validation analysis. To appraise the downstream effects of miR-19a/b-3p, we selected 82 potential target genes for further analysis using high throughput RT-qPCR. These target genes were selected by cross-match between the potential target genes predicted by miRWalk (3,066 potential target genes), testis-specific genes annotated in Human Protein Atlas (2,237 protein-coding genes), and the testis-specific genes that are related to spermatogenesis and male infertility as identified by the ToppGene Suite algorithm (Chen et al., 2009). The overlap of genes (130 target genes identified) was further filtered according to their functional role in spermatogenesis and sperm function, context score of mRNA-miRNA interaction, their binding site within the 3′UTR of the transcript, and seed sequence (7mer, 8mer). A total of 53 genes were significantly confirmed as downstream targets, including 51 target genes with lower expression and two genes with higher expression in sperm samples collected from oligoasthenozoospermic men compared to normozoospermic men. Taking into consideration the simultaneous higher expression of miR-19a/b-3p and lower expression of 51 target genes, we can assume a direct interaction between miRNA at the binding site in the 3′UTR of the target genes. Identification of spermatogenesis dysfunctions is usually done through the evaluation of the end product of the process; thus, sperm count, motility and morphology are typically the means of determining whether the spermatogenesis process is being completed as it should (excluding men with obstructive azoospermia). Even a small interruption of the spermatogenesis process can create major difficulties for couples attempting to conceive. Therefore, without proper spermatogenesis occurring, a male human will be subfertile or infertile. In our study, the identified higher miRNAs and lower mRNAs expression levels may lead to male subfertility and/or infertility. However, it is remarkable, that our studied miRNAs and their experimentally identified mRNA targets have previously been reported to play an essential role in the early stages of testicular differentiation and development, including a functional role in sperm motility and morphology.

A close look into the dysregulated target genes, some genes play a wide variety of functions within the testes, some other genes have an unknown or poorly characterized function and some other target genes are involved in the basal cellular processes without any clear function in spermatogenesis or infertility. The aberrant expression of some, if not most of these gene within the testis may disrupt some important cellular processes and ultimately lead to male subfertility or in worse cases lead to infertility and/or sterility. Furthermore, some other genes have functions that have been clarified only in model organisms, especially in mice. We argue if these genes could potentially show similar effects in humans. Based on previous studies, many genes were found to be associated with infertility or are dysregulated in various phenotypes, but it is still not clear their exact function in the male infertility. For example, *FAM104A* was down-regulated in men with non-obstructive azoospermia (NOA) (Hu et al., 2021), and *TMEM215* was up-regulated in spermatozoa of infertile men (Cheung et al., 2019) as determined by microarray and sequencing analyses, respectively. *MCHR2* has been recognized as one of 371 clinically relevant genes for infertility as determined by high-resolution chromosome ideograms analysis (Butler et al., 2016).

In our study, we reported several lower expressed genes that are associated with the early stages of sperm production during the spermatogenesis process. Of these target genes, *SPATA12* is one of the most well-known genes involved in the regulation of spermatogenesis and is mainly expressed in spermatocytes, spermatids, and spermatozoa (Dan et al., 2007). The amount of these precursor and mature cells were directly correlated with the expression level of *SPATA12* (Dan et al., 2007). The *UBQLNL* gene encodes Ubiquilin and is particularly active in the post-meiotic stages of spermatogenesis (Marin, 2014). Nevertheless, little is known about the exact function of *UBQLNL* in male fertility (Marin, 2014). Another spermatogenesis-associated gene is *ELAVL2* which regulates the proliferation of spermatogonia and apoptosis (Yang et al., 2021). *ELAVL2* is conserved in spermatogonia stem cells (SSCs) and was down-regulated in NOA (Yang et al., 2021). SPIRE1 acts as a regulator of ectoplasmic specialization to modulate spermatogenesis by supporting germ cell development (Wen et al., 2018). Moreover, *SPIRE1* supports epithelial and endothelial cell function by the initiation of actin nucleation for building actin microfilaments efficiently (Wen et al., 2018). Of the target genes that were differentially down-regulated in sperm of subfertile as compared to fertile controls and play a role in the meiosis stage of spermatogenesis is *SCML2*. It is expressed in both gonads, and functions as a regulator of heterochromatin after the meiosis stage during spermatogenesis (Maezawa et al., 2018). *SCML2* has globally suppressed the somatic gene expression in late spermatogenesis, during meiosis, and into post-meiotic stages, while a distinct class of late spermatogenesis genes, is being activated (Hasegawa et al., 2015; Sin et al., 2015). *HSPA2* plays a key regulator role in the sperm maturation within the testis and capacitation in the reproductive tract of females. Additionally, *HSPA2* is a member of the 70 kDa heat shock protein family, that is, associated with the IVF outcome (Nixon et al., 2015b). Its main function is to support the folding, transport, and formation of protein complexes (Nixon et al., 2015b). A reduction of its expression leads to complications with sperm-egg recognition and fertilization, as reviewed in Nixon *et al.* (Nixon et al., 2015b). The reduction of HSPA2 protein expression level was observed in seminal plasma samples of men with oligozoospermia as compared to the controls (Nowicka-Bauer et al., 2022), suggesting the possibility of using *HSPA2* genes as a biomarker for spermatogenesis status, especially in cryptozoospermic males, and as a predictive biomarker for the success of assisted reproductive techniques (ART) (Nowicka-Bauer et al., 2022). Moreover, the expression of the *HSPA2* gene was down-regulated in human testes with abnormal spermatogenesis (Son et al., 2000). These findings indicate that these genes are potentially required for the completion of normal spermatogenesis and their loss of function in the case of aberrant expression could lead to a reduction of fecundity.

A portion of our identified lower expressed genes are not yet associated with the maturation of sperm but have structural functions and their absence may reduce sperm motility. *ODF4* gene, for example, encodes the outer dense fibers of the sperm tail, that surround the axoneme and protect the elastic rigidity of the sperm flagellum (Cao et al., 2006). The down-regulation of *ODF4* might result in abnormal sperm structure and motility. Another identified gene, DNAI1 originates from a dynein family, which is responsible for the cilia movement. Mutation of this gene leads to ciliary dyskinesia and male infertility (Knowles et al., 2012; Pereira et al., 2015; Precone et al., 2020). A reduction in the *DNAI1* gene might lead to ciliary loss of function, resulting in immotile sperm and ultimately infertility. Like *ODF4* and *DNAI1*, the absence of *STK33* leads to reduced motility and morphological changes in the flagellum. The encoded serine/threonine kinase seems to play a crucial role in the organization of flagellar ultrastructure (Ma et al., 2021). *AQP5* is one of the aquaporin family and is required for cell volume, by regulating the permeability of the cell membrane (Liu et al., 2006). In sperm, aquaporins protect cells from harmful swelling to maintain normal sperm function and survival (Chen and Duan, 2011). Downregulation of *AQP5*, as shown in our results, might decrease sperm function and could lead to apoptosis. In addition to spermatogenesis and structural properties, hormonal regulation also plays a major role in the development of infertility. Our results indicated that *FSHR* is down-regulated in patients with oligoasthenozoospermia. *FSHR* functions as a receptor of follicle-stimulating hormone (FSH), which stimulates several pathways to control the trophic and mitotic effects of Sertoli cells (Casarini et al., 2020). Previous studies indicated that a loss of FSHR reduced the FSH-dependent intracellular signaling (Casarini et al., 2014) and the volume of testes (Grigorova et al., 2013). Therefore, we assume that a reduction in FSHR expression might reduce the fertility of men.

In summary, we would like to point out that this study has also a number of limitations. The major limitation is the lack of miRNA-target interaction validation experiments to elucidate the complex downstream effects of miR-19a/b-3p and to lay the ground for an experimentally validated network in sperm. For the validation procedure, the Luciferase-Assay was globally used to confirm the miRNA binding site within the 3′UTR regions of the target gene. Experimentally, transfection of the human embryonic kidney cell line (HEK-293T) with both, the expression vector (pSG5), which includes the precursor miRNA of interest, and the reporter plasmid (pMiR-RNL-TK), which encodes the binding site of the miRNA within the 3′UTR of the target gene. The direct interactions between the over-expressed miRNA (miR-19a/b-3p) and 3′UTR of mRNAs (significant 51 target genes with lower expression levels), down-regulate the firefly luciferase protein resulting in the reduction of luminescence that will be relatively quantified. In our study, we were unable to produce the expression plasmid, that has the ability to over-expresses the miR-19a-3p and/or miR-19b-3p. We designed a variety of plasmid inserts, which encodes the precursor miRNAs with 50 bp up and downstream, the sequence which forms the hairpin structure, only the sequence of precursor, the full length 17–92 cluster, and the miR-19b precursor sequence with the surrounding miRNA of the cluster (data not shown). Furthermore, the precise miRNA-target interaction validation experiments should be evaluated in human germ cell-specific cell lines, not HEK-293T, However, human germ cell line has not yet been available on the market. Nevertheless, consistent with the idea of post-transcriptional down-regulation of mRNA while miRNA is over-expressed at the same time, our results give us, however, an indication of the complex downstream effects of miR-19a/b-3p and their target genes. The outcome of our study provides further insights into the miRNA-target networks and might contribute to a deeper understanding of idiopathic infertility and the future use of miRNA as a biomarker for infertility.

## Data Availability

The original contributions presented in the study are included in the article/Supplementary Material, further inquiries can be directed to the corresponding author.
