# Supplementary material for: MicroRNA-targeting in male infertility: Sperm microRNA-19a/b-3p and its spermatogenesis related transcripts content in men with oligoasthenozoospermia
Source: Front Cell Dev Biol. 2022 Sep 21;10:973849. doi: 10.3389/fcell.2022.973849 (PMC9533736; doi:10.3389/fcell.2022.973849)
Supplement: Supplementary file 1 [file Table1.DOCX]

**Supplementary Table 1:** The primers and design RefSeq of the genes included in the study.

| **Target** | **Forward Primer** | **Reverse Primer** | **Design RefSeq** | **Results** |
| --- | --- | --- | --- | --- |
| AQP5 | ACTTCACTGGCTGCTCCA | GATGGGCCCTACCCAGAAA | NM_001651.N | Significant |
| ASAP2 | CTGACCAACAAAGGCCAACC | ACCATAGCATTGGACAGAGGAC | NM_003887.N | Significant |
| ATF7IP2 | TGGCAGTCATCACTTGACAC | GCTCTGAAGTTTTCATGCGTTTAA | NM_001352120.N | Significant |
| BOD1L2 | TCCAGCAAACGACAACCAAC | TGTCCACTCCAGCTTCTGAC | NM_001257964.N | Significant |
| BRCA2 | AGAGCCGATTACCTGTGTACC | TCTTGACCAGGTGCGGTAAA | NM_000059.N | Significant |
| C22ORF31 | TCCCCTTACTAAGCTAGCGAAA | CTGGGGTCTCGTCTCACA | NM_015370.N | Significant |
| C2ORF42 | CTGTGGTTGCTTCCTCGTTAAA | CCAGCCAGTCTTGGAAGGATA | NM_001348758.N | Significant |
| CCDC87 | ACTGAATCAGCCACCCTCAC | ACGGTAGACGTTGGGACTAGTA | NM_018219.N | Significant |
| CCER1 | AACCACCCGTGTGCTCTAA | GCAGGGGAATTTCCAGAATCC | NM_152638.N | Significant |
| COX8C | AGCACTGGAGCTTGCGTTA | ATCGCGTCAGGTGAGACAAA | NM_182971.N | Significant |
| CPEB1 | CCCAAAGACCCCTTCAGCATA | AGGTACAGGTGGCTTCATTCA | NM_001365240.N | Significant |
| CSMD1 | AGCAAAACCAGGCCACTCTA | CTCTGACCTCCAAGGTGTCATAA | NM_033225.N | Significant |
| CSNK1G1 | TTGACCTCTGTGACCGAACA | GTGCACGTATTCCATTCGAGAA | NM_022048.N | Significant |
| DCAF12L1 | AACAAGGTGTTCGCGTCAC | CCACCACGAAAAGCGTGTTA | NM_178470.N | Significant |
| DDHD1 | CCGCCGACACTATGGAGAA | TCCAAGTCCCTTTCCAATGCTA | NM_001160148.N | Significant |
| DEPDC1 | TATCAAAGGGAGGTGGGGATCA | TAAGTGGCGAAGTTGCAGGAA | NM_001114120.N | Significant |
| DNAI1 | GGGAAGATGGCCATGAGGAA | TGATCTTAGCAGCTTGGGACAA | NM_001281428.N | Significant |
| DPYSL5 | GAGGGACCTTCACGAATCCA | GCTGAAGCTCGCTTTGGAA | NM_020134.N | Significant |
| ELAVL2 | AGAGAAAGCTATCAACACCCTGAA | AACTTGGGCGAGCATAGGAA | NM_001351455.N | Significant |
| FAM104A | CCCACTTCCACAGCCTACA | TCCCTTCACAGAAGGCAAGAA | NM_001098832.N | Significant |
| FAM169A | AGGAGAGGCCATTGGGTTTTA | CTGGCAATTGGTAACTTTGGGTA | NM_015566.N | Significant |
| FHL5 | CCACTGCAAGAGGACCATCA | CAGGTTTCATGCCAGTAGTTTCC | NM_020482.N | Significant |
| FSHR | GATGTGTTCTCCAACCTTCCC | AAGGCCTCAGGGTTGATGTA | NM_000145.N | Significant |
| GNAT1 | ACCACTGGCATCATCGAGAC | CCCGCCCACATCGAACA | NM_144499.N | Significant |
| GOLGA6A | GCAGTTGGAGCAGCAAGTAAA | GGTGGCTGGCAGCTTCTA | NM_001038640.N | Significant |
| GOLGA6D | ATGAGCGCTGCACTATGGTA | GCTGCAGCTCATGATGCAATA | NM_001145224.N | Significant |
| HSF5 | TACACACCCTCAGCACAGTA | TCCATGTGGGTAGGAGAACA | NM_001080439.N | Significant |
| HSPA2 | GTGTCATGACCCCACTCATCA | GTTGTCCGAGTAGGTGGTGAA | NM_021979.N | Significant |
| MCHR2 | AATTTGCCTGTAGTGCCATCA | CTCCAACGTGTCAGTCGAAA | NM_001040179.N | Significant |
| MED26 | CCAGCCTGGAGAAATACCCTA | ACGTCGTTGATGAGCTTCC | NM_004831.N | Significant |
| MYBL1 | CCTTGTGCAGCTATGGATCA | CAGGTGACACATACTGATACCC | NM_001080416.N | Significant |
| ODF4 | GTTGGCTGGTGCTTATCCTA | GTGGCTCAGAATCAGACTCC | NM_153007.N | Significant |
| PCDHA7 | TATTCCAGAGGACGCCCAAC | ACCTGTCCGTTGACTCCAAA | NM_018910.N | Significant |
| POC1A | CGGGAATGTGTCCACTCGTA | GTGGAAGTCCACATAGGTGACA | NM_001161581.N | Significant |
| PRSS54 | ACACCACCAGTCTTGCAGAA | TCTGGAGTTTGTATAGGGGACAC | NM_001080492.N | Significant |
| REEP1 | GTCAAATGGATGATGTACTGGATTA | CAACAAAGGAAGATGTCTGTGAA | NM_001371279.N | Significant |
| RFX4 | TGGCTGGATACCATGGTTGAC | TGCTGGGCCACTTTCTTCAA | NM_213594.N | Significant |
| SAMD4A | CTTTCCCTCGGAAAACCCTTCTA | AGGGAGTTGGATTGCCCAAA | NM_015589.N | Significant |
| SCML2 | TCGCTTCTGGGCCATGTAA | CAGATGAGGGCCAAAGTTTCC | NM_006089.N | Significant |
| SENP8 | GAGCAACTCAGTTCACGCAAA | CACAAAGGCCAGTTTGTCTCC | NM_001172111.N | Significant |
| SPATA12 | GCCTGCAGTATCAGACCTCAA | TGTCCACTTTCACAGCTTCCC | NM_181727.N | Significant |
| SPIRE1 | GAGCGATGAATCTAGCACAGAC | CCTCATCACCTGTACCCAGAA | NM_001128626.N | Significant |
| STK33 | ACTATAGCCAGCAGTGTGAC | AGGGTGGTTCTCCACGTA | NM_001352398.N | Significant |
| TDRD10 | GGTGTATGTTGGCAATCTTCC | ACATCAAGAGGGTTGAAGTCC | NM_001098475.N | Significant |
| TKTL2 | AGTGCCTCAACGTGGGAAAA | CGGCTGCTATAATGTGTCTGGTA | NM_032136.N | Significant |
| TMEM215 | TGGAGAGGGCAGGAAACC | CGCATGTTTCACTCCATCCA | NM_212558.N | Significant |
| TTLL2 | ACCTGTGTTCCTCCACACAA | CCTCGGATGGAATGTTAAGGGTA | NM_031949.N | Significant |
| UBN2 | TAAGGAAAGCCGGAGTGTTCA | TGGGTTTAGGTGCAGGAATCA | NM_173569.N | Significant |
| UBQLNL | TGGCAGGACAAGTGCTAGAA | GTGAGCTGGTCTTGTTGTTCC | NM_145053.N | Significant |
| USP6 | GCCACTACCAATGGACAGTTAC | TAGTCCATACCGTACAGGGGTA | NM_001304284.N | Significant |
| ZNF280B | CGGCAGCTCGCGAACA | CTCAGGTTTATTACAACGCAACTTTTAA | NM_080764.N | Significant |
| WNK3 | TTTCACAACCCCAGGTTTCC | AGCTCCAACTGGTTGGGATA | NM_020922.N | Significant |
| MBNL3 | GCCCAAAACTGATGCGTTCA | CAATCATTCTCCCCACGGGTA | NM_001170704.N | Significant |
| RALGPS2 | AGATGACACCTTGCATTCCCTA | TGCTGCCAGTTGATGGGTA | NM_001286247.N | Borderline significant |
| ZNF711 | CCCGAAGGTATGAAGATTGTCA | AAGGTACTGTGCTGCTGTAC | NM_001330574.N | Borderline significant |
| ZPLD1 | TGCTCAGTTCAACGGCTACA | TAGCCTGCACTCCACAATAGAC | NM_001329788.N | Borderline significant |
| SFMBT1 | GGGTGGTGTGAGCAGAATAA | GCAGAAACTCATCCCAGTCA | NM_016329.N | Borderline significant |
| ARRDC5 | GGAACTTCCACCTTCCACAA | GGCAGCAGTTGTAGGAGAC | NM_001367189.N | Non-Significant |
| C5ORF49 | GACCCCAAAGAGCACAGCTA | TGATCATAATCTAGGCGCCTCTTA | NM_001089584.N | Non-Significant |
| CCDC15 | TCCTGATACCTGTGCCAACAA | TGAATGAATGTAGTGCCCGAGTA | NM_025004.N | Non-Significant |
| CEP170 | ACAGGCTGCCACGAGAAA | GCGTGTTGCTTATCCACACTAC | NM_014812.N | Non-Significant |
| CEP55 | GGAGCTCCGAAAAGCAAGAA | TGGCTCTGTGATGGCAAAC | NM_018131.N | Non-Significant |
| CLEC18B | ACCTGGGCAGAGGTGTG | CAAGAAACTCTCCTTCCTGTTCAG | NM_001011880.N | Non-Significant |
| CLHC1 | TGCAGCAAACAGTCCTAGAA | AATGGAAGAGGCTTTCCTCTAA | NM_001353781.N | Non-Significant |
| DNAJC27 | AGCTTGGACCTCATGGAAAC | ACACAGCGATGTTTGGTACA | NM_016544.N | Non-Significant |
| INHBB | GAGCGCGTTTCCGAAATCA | TGCCTTCGTTGGAGATGAAGAA | NM_002193.N | Non-Significant |
| LRP2BP | GGGCATCACCAGGGATGAAA | ATCTGCCAATGCGGGATTCA | NM_018409.N | Non-Significant |
| PRDM9 | GCAGCCAACAATGGATACTCC | ATCCAGTTGGCCCAGGATTTA | NM_020227.N | Non-Significant |
| RPL39L | AGCATCTTTGCCTCTTTGGAGTA | ACATGGCGAGAAACAGAGTCA | NM_052969.N | Non-Significant |
| TDRD6 | GCAGTGATGGAAGCAAGCA | GACCACACAACACATTTAGATCCA | NM_001010870.N | Non-Significant |
| WIPF3 | GAGGTCAACTGCGAAATGGAA | CTTCCACAGAATGGAACGTGAA | NM_001080529.N | Non-Significant |
| ZMYND12 | ACTATGAAGAGGCCCGTTATCA | GGTGGAAGTAGCCTCCTGAA | NM_001146192.N | Non-Significant |
| SH2D6 | AGGAAGGAAATCGTCTCTTCCC | CTGGGGCGCACGGTATA | NM_201594.N | Omitted |
| TCL1B | TGGTCGTGCGGTTCAATCC | AGGAGAGTAGCTCCCGGGTA | NM_004918.N | Omitted |
| CALCR | CCATCTACTGCTTCTGCAACA | CAACGCTGGTTCCACTGAA | NM_001164737.N | Omitted |
| EPHA10 | GGACTGCACTGCCAAGTAA | GCACATTGCACACTTGGTAC | NM_001099439.N | Omitted |
| FAM9A | GCCGCTACTTGAGCAATTAC | CTTCACTGGAAGAAATGATGCA | NM_001171186.N | Omitted |
| GABRR1 | GCCTGATGGGAAAGTGCTCTA | TCCAAGGGAAATCGGCTGAA | NM_001256704.N | Omitted |
| MAGEA10 | CAGGCTTGAGATCGGCTGAA | CGACCCACTGTGTCTGTAGAA | NM_001011543.N | Omitted |
| NYAP1 | AAGGTCCTCTATGGAGGGAGAA | TCGGCACTGCCATTCCA | NM_173564.N | Omitted |
| POU4F1 | TCACTTTGCCATGCATCCC | GGCGAAGAGGTTGCTCTG | NM_006237.N | Omitted |
| SCN9A | GGTGTTGGCTGGATTTCCTA | ATGGGGCCAAGATCTGAGTA | NM_002977.N | Omitted |
| ACTB | CCAACCGCGAGAAGATGAC | TAGCACAGCCTGGATAGCAA | NM_001101.N | Endogenous Control |
| GAPDH | GAGAACGGGAAGCTTGTCATCA | TGGACTCCACGACGTACTCA | NM_002046. | Endogenous Control |

**Supplementary Table 2:** Correlation analysis between the expression level of genes and basic semen parameters.

| **Gene** | **Count (106/mL)** | | | **Motility (% motile)** | | | **Morphology (%)** | | |
| --- | --- | --- | --- | --- | --- | --- | --- | --- | --- |
|  | **r** | **95% confidence interval** | **P value** | **r** | **95% confidence interval** | **P value** | **r** | **95% confidence interval** | **P value** |
| AQP5 | 0.34 | 0.527 to 0.125 | 0.0019 | 0.36 | 0.543 to 0.147 | 0.0010 | 0.29 | 0.488 to 0.073 | 0.0081 |
| ASAP2 | 0.34 | 0.52 to 0.122 | 0.0020 | 0.39 | 0.561 to 0.179 | 0.0003 | 0.28 | 0.471 to 0.0571 | 0.0118 |
| ATF7IP2 | **0.48** | 0.634 to 0.286 | <0.0001 | **0.43** | 0.598 to 0.231 | <0.0001 | 0.32 | 0.508 to 0.106 | 0.0032 |
| BOD1L2 | **0.45** | 0.611 to 0.25 | <0.0001 | **0.47** | 0.628 to 0.276 | <0.0001 | 0.32 | 0.508 to 0.105 | 0.0033 |
| BRCA2 | 0.21 | 0.44 to 0.0537 | 0.1082 | 0.25 | 0.472 to 0.013 | 0.0553 | 0.27 | 0.494 to 0.0162 | 0.0325 |
| C22ORF31 | 0.34 | 0.527 to 0.128 | 0.0017 | 0.31 | 0.499 to 0.0914 | 0.0049 | 0.25 | 0.453 to 0.031 | 0.0222 |
| C2ORF42 | 0.32 | 0.511 to 0.109 | 0.0029 | 0.36 | 0.539 to 0.147 | 0.0009 | 0.26 | 0.453 to 0.0345 | 0.0204 |
| CCDC87 | **0.42** | 0.584 to 0.211 | 0.0001 | 0.39 | 0.563 to 0.181 | 0.0003 | 0.34 | 0.524 to 0.128 | 0.0017 |
| CCER1 | **0.42** | 0.587 to 0.216 | <0.0001 | 0.38 | 0.552 to 0.165 | 0.0005 | 0.33 | 0.517 to 0.118 | 0.0023 |
| COX8C | **0.43** | 0.594 to 0.226 | <0.0001 | 0.39 | 0.563 to 0.182 | 0.0003 | 0.32 | 0.51 to 0.109 | 0.0030 |
| CPEB1 | 0.32 | 0.507 to 0.096 | 0.0045 | 0.38 | 0.557 to 0.164 | 0.0006 | 0.28 | 0.473 to 0.0511 | 0.0141 |
| CSMD1 | 0.37 | 0.546 to 0.154 | 0.0008 | **0.44** | 0.602 to 0.234 | <0.0001 | 0.36 | 0.541 to 0.148 | 0.0010 |
| CSNK1G1 | 0.37 | 0.548 to 0.16 | 0.0006 | **0.43** | 0.596 to 0.228 | <0.0001 | 0.30 | 0.49 to 0.0818 | 0.0063 |
| DCAF12L1 | **0.42** | 0.59 to 0.219 | <0.0001 | 0.37 | 0.551 to 0.165 | 0.0005 | 0.32 | 0.507 to 0.105 | 0.0034 |
| DDHD1 | 0.27 | 0.467 to 0.0516 | 0.0135 | 0.32 | 0.509 to 0.106 | 0.0032 | 0.22 | 0.423 to 0.0031 | 0.0468 |
| DEPDC1 | 0.27 | 0.468 to 0.0539 | 0.0128 | 0.27 | 0.465 to 0.0497 | 0.0142 | 0.21 | 0.41 to 0.0181 | 0.0635 |
| DNAI1 | 0.35 | 0.532 to 0.129 | 0.0018 | 0.35 | 0.536 to 0.135 | 0.0015 | 0.25 | 0.448 to 0.0188 | 0.0295 |
| DPYSL5 | **0.45** | 0.608 to 0.244 | <0.0001 | **0.52** | 0.668 to 0.336 | <0.0001 | **0.42** | 0.589 to 0.216 | <0.0001 |
| ELAVL2 | 0.36 | 0.543 to 0.15 | 0.0009 | 0.38 | 0.557 to 0.17 | 0.0005 | 0.27 | 0.464 to 0.0458 | 0.0157 |
| FAM104A | **0.42** | 0.592 to 0.222 | <0.0001 | **0.40** | 0.568 to 0.188 | 0.0002 | 0.32 | 0.508 to 0.106 | 0.0032 |
| FAM169A | 0.25 | 0.447 to 0.0263 | 0.0247 | 0.30 | 0.49 to 0.0818 | 0.0063 | 0.22 | 0.421 to 0.00518 | 0.0488 |
| FHL5 | 0.28 | 0.474 to 0.0581 | 0.0117 | 0.28 | 0.478 to 0.0636 | 0.0101 | 0.19 | 0.393 to 0.041 | 0.0978 |
| FSHR | 0.37 | 0.603 to 0.0682 | 0.0146 | **0.43** | 0.651 to 0.145 | 0.0035 | 0.25 | 0.513 to 0.0641 | 0.1077 |
| GNAT1 | 0.27 | 0.487 to 0.0313 | 0.0235 | **0.42** | 0.604 to 0.196 | 0.0004 | 0.36 | 0.553 to 0.121 | 0.0029 |
| GOLGA6A | 0.29 | 0.481 to 0.0675 | 0.0092 | 0.34 | 0.522 to 0.122 | 0.0021 | 0.26 | 0.457 to 0.0369 | 0.0194 |
| GOLGA6D | **0.44** | 0.604 to 0.241 | <0.0001 | **0.42** | 0.588 to 0.218 | <0.0001 | 0.36 | 0.541 to 0.15 | 0.0009 |
| HSF5 | 0.35 | 0.536 to 0.14 | 0.0012 | 0.36 | 0.543 to 0.15 | 0.0009 | 0.25 | 0.449 to 0.0262 | 0.0248 |
| HSPA2 | **0.43** | 0.595 to 0.228 | <0.0001 | 0.38 | 0.56 to 0.176 | 0.0004 | 0.32 | 0.504 to 0.0996 | 0.0038 |
| MCHR2 | **0.47** | 0.682 to 0.177 | 0.0021 | **0.48** | 0.692 to 0.194 | 0.0015 | 0.19 | 0.476 to 0.136 | 0.2398 |
| MED26 | **0.41** | 0.578 to 0.202 | 0.0001 | **0.48** | 0.631 to 0.281 | <0.0001 | 0.37 | 0.544 to 0.154 | 0.0008 |
| MYBL1 | 0.35 | 0.532 to 0.132 | 0.0016 | 0.39 | 0.565 to 0.178 | 0.0004 | 0.28 | 0.478 to 0.0607 | 0.0110 |
| ODF4 | 0.27 | 0.469 to 0.0401 | 0.0184 | 0.18 | 0.398 to 0.0483 | 0.1089 | 0.17 | 0.386 to 0.0616 | 0.1366 |
| PCDHA7 | 0.38 | 0.553 to 0.167 | 0.0005 | 0.35 | 0.531 to 0.136 | 0.0013 | 0.32 | 0.507 to 0.104 | 0.0034 |
| POC1A | 0.32 | 0.509 to 0.0975 | 0.0043 | 0.33 | 0.52 to 0.112 | 0.0028 | 0.36 | 0.539 to 0.139 | 0.0013 |
| PRSS54 | 0.34 | 0.528 to 0.121 | 0.0023 | 0.38 | 0.562 to 0.168 | 0.0006 | 0.30 | 0.497 to 0.0788 | 0.0072 |
| REEP1 | 0.38 | 0.558 to 0.174 | 0.0004 | 0.36 | 0.542 to 0.152 | 0.0008 | 0.31 | 0.497 to 0.0909 | 0.0049 |
| RFX4 | 0.38 | 0.556 to 0.171 | 0.0004 | **0.45** | 0.609 to 0.248 | <0.0001 | 0.34 | 0.526 to 0.129 | 0.0016 |
| SAMD4A | 0.39 | 0.562 to 0.179 | 0.0003 | **0.44** | 0.605 to 0.242 | <0.0001 | 0.30 | 0.491 to 0.0831 | 0.0060 |
| SCML2 | 0.34 | 0.562 to 0.0816 | 0.0094 | 0.28 | 0.513 to 0.0118 | 0.0358 | 0.28 | 0.515 to 0.0146 | 0.0340 |
| SENP8 | **0.45** | 0.608 to 0.247 | <0.0001 | **0.44** | 0.604 to 0.241 | <0.0001 | 0.37 | 0.548 to 0.159 | 0.0006 |
| SPATA12 | 0.37 | 0.548 to 0.16 | 0.0006 | **0.40** | 0.574 to 0.197 | 0.0002 | 0.25 | 0.451 to 0.0319 | 0.0217 |
| SPIRE1 | 0.34 | 0.521 to 0.123 | 0.0020 | 0.38 | 0.559 to 0.175 | 0.0004 | 0.26 | 0.454 to 0.0354 | 0.0200 |
| STK33 | 0.28 | 0.477 to 0.0557 | 0.0126 | 0.27 | 0.465 to 0.041 | 0.0178 | 0.15 | 0.365 to 0.0801 | 0.1869 |
| TDRD10 | 0.36 | 0.537 to 0.142 | 0.0011 | 0.39 | 0.564 to 0.18 | 0.0003 | 0.30 | 0.489 to 0.0775 | 0.0071 |
| TKTL2 | **0.42** | 0.587 to 0.215 | <0.0001 | 0.39 | 0.562 to 0.18 | 0.0003 | 0.34 | 0.527 to 0.131 | 0.0016 |
| TMEM215 | 0.39 | 0.562 to 0.176 | 0.0004 | **0.42** | 0.592 to 0.22 | <0.0001 | 0.29 | 0.481 to 0.0678 | 0.0091 |
| TTLL2 | 0.37 | 0.552 to 0.16 | 0.0007 | 0.39 | 0.563 to 0.176 | 0.0004 | 0.31 | 0.498 to 0.0865 | 0.0057 |
| UBN2 | 0.29 | 0.478 to 0.0663 | 0.0094 | 0.37 | 0.545 to 0.156 | 0.0007 | 0.25 | 0.447 to 0.0266 | 0.0245 |
| UBQLNL | **0.44** | 0.606 to 0.244 | <0.0001 | **0.44** | 0.602 to 0.238 | <0.0001 | 0.35 | 0.532 to 0.138 | 0.0012 |
| USP6 | 0.31 | 0.504 to 0.094 | 0.0046 | 0.33 | 0.514 to 0.108 | 0.0032 | 0.25 | 0.447 to 0.0207 | 0.0281 |
| ZNF280B | 0.35 | 0.534 to 0.137 | 0.0013 | **0.40** | 0.576 to 0.197 | 0.0002 | 0.29 | 0.479 to 0.0651 | 0.0098 |
|  |  |  |  |  |  |  |  |  |  |
| **WNK3** | **-0.32** | **-0.0845 to -0.521** | **0.0070** | **-0.44** | **-0.224 to -0.618** | **0.0001** | **-0.40** | **-0.18 to -0.588** | **0.0005** |
| **MBNL3** | **-0.33** | **-0.0985 to -0.523** | **0.0047** | **-0.34** | **-0.113 to -0.534** | **0.0032** | **-0.27** | **-0.0385 to -0.478** | **0.0196** |

- Spearman correlation analysis
- An Unpaired two-tailed t-test was used to calculate the p-value.
- Significant change in abundance level was considered with a p-value < 0.05

**Supplementary Table 3:** Correlation analysis between the expression level of miRNAs and target genes

| **Gene** | **miR-19a-3p** | | | **miR-19-b-3p** | | |
| --- | --- | --- | --- | --- | --- | --- |
|  | **r** | **95% confidence interval** | **P value** | **r** | **95% confidence interval** | **P value** |
| AQP5 | -0.41 | -0.579 to -0.199 | 0.0002 | -0.29 | -0.48 to -0.0636 | 0.0103 |
| ASAP2 | -0.47 | -0.627 to -0.275 | <0.0001 | -0.40 | -0.571 to -0.192 | 0.0002 |
| ATF7IP2 | -0.49 | -0.645 to -0.303 | <0.0001 | -0.34 | -0.521 to -0.122 | 0.002 |
| BOD1L2 | -0.53 | -0.674 to -0.349 | <0.0001 | -0.39 | -0.565 to -0.185 | 0.0003 |
| BRCA2 | -0.33 | -0.539 to -0.077 | 0.0094 | -0.14 | -0.383 to 0.121 | 0.2778 |
| C22ORF31 | -0.47 | -0.629 to -0.276 | <0.0001 | -0.37 | -0.55 to -0.16 | 0.0006 |
| C2ORF42 | -0.45 | -0.612 to -0.253 | <0.0001 | -0.35 | -0.53 to -0.135 | 0.0014 |
| CCDC87 | -0.49 | -0.64 to -0.295 | <0.0001 | -0.37 | -0.549 to -0.162 | 0.0006 |
| CCER1 | -0.46 | -0.616 to -0.259 | <0.0001 | -0.35 | -0.531 to -0.136 | 0.0013 |
| COX8C | -0.47 | -0.623 to -0.269 | <0.0001 | -0.37 | -0.548 to -0.16 | 0.0006 |
| CPEB1 | -0.44 | -0.61 to -0.241 | <0.0001 | -0.35 | -0.532 to -0.13 | 0.0017 |
| CSMD1 | -0.51 | -0.656 to -0.318 | <0.0001 | -0.40 | -0.577 to -0.198 | 0.0002 |
| CSNK1G1 | -0.50 | -0.651 to -0.312 | <0.0001 | -0.42 | -0.584 to -0.211 | 0.0001 |
| DCAF12L1 | -0.46 | -0.619 to -0.262 | <0.0001 | -0.36 | -0.54 to -0.15 | 0.0009 |
| DDHD1 | -0.43 | -0.597 to -0.23 | <0.0001 | -0.35 | -0.527 to -0.132 | 0.0015 |
| DEPDC1 | -0.47 | -0.63 to -0.279 | <0.0001 | -0.42 | -0.588 to -0.218 | <0.0001 |
| DNAI1 | -0.42 | -0.589 to -0.21 | 0.0001 | -0.34 | -0.522 to -0.116 | 0.0026 |
| DPYSL5 | -0.55 | -0.692 to -0.375 | <0.0001 | -0.41 | -0.584 to -0.209 | 0.0001 |
| ELAVL2 | -0.48 | -0.634 to -0.283 | <0.0001 | -0.36 | -0.537 to -0.142 | 0.0012 |
| FAM104A | -0.44 | -0.603 to -0.239 | <0.0001 | -0.31 | -0.501 to -0.0961 | 0.0042 |
| FAM169A | -0.39 | -0.562 to -0.18 | 0.0003 | -0.31 | -0.497 to -0.0912 | 0.0049 |
| FHL5 | -0.40 | -0.576 to -0.197 | 0.0002 | -0.28 | -0.473 to -0.0566 | 0.0121 |
| FSHR | -0.43 | -0.647 to -0.14 | 0.0039 | -0.25 | -0.515 to 0.0604 | 0.1026 |
| GNAT1 | -0.44 | -0.615 to -0.212 | 0.0002 | -0.24 | -0.46 to 0.00319 | 0.0466 |
| GOLGA6A | -0.50 | -0.653 to -0.313 | <0.0001 | -0.43 | -0.597 to -0.227 | <0.0001 |
| GOLGA6D | -0.47 | -0.627 to -0.275 | <0.0001 | -0.37 | -0.545 to -0.156 | 0.0007 |
| HSF5 | -0.45 | -0.616 to -0.255 | <0.0001 | -0.34 | -0.521 to -0.12 | 0.0022 |
| HSPA2 | -0.47 | -0.626 to -0.273 | <0.0001 | -0.37 | -0.551 to -0.164 | 0.0005 |
| MCHR2 | -0.43 | -0.654 to -0.128 | 0.0054 | -0.33 | -0.586 to -0.0166 | 0.0345 |
| MED26 | -0.53 | -0.669 to -0.341 | <0.0001 | -0.41 | -0.584 to -0.211 | 0.0001 |
| MYBL1 | -0.46 | -0.623 to -0.264 | <0.0001 | -0.32 | -0.51 to -0.103 | 0.0037 |
| ODF4 | -0.24 | -0.443 to -0.00723 | 0.0378 | -0.12 | -0.337 to 0.118 | 0.3168 |
| PCDHA7 | -0.41 | -0.579 to -0.204 | 0.0001 | -0.32 | -0.504 to -0.1 | 0.0038 |
| POC1A | -0.47 | -0.63 to -0.272 | <0.0001 | -0.35 | -0.531 to -0.127 | 0.0019 |
| PRSS54 | -0.35 | -0.536 to -0.132 | 0.0017 | -0.26 | -0.461 to -0.032 | 0.0221 |
| REEP1 | -0.38 | -0.555 to -0.17 | 0.0005 | -0.30 | -0.493 to -0.086 | 0.0056 |
| RFX4 | -0.50 | -0.651 to -0.313 | <0.0001 | -0.39 | -0.561 to -0.178 | 0.0003 |
| SAMD4A | -0.50 | -0.649 to -0.309 | <0.0001 | -0.39 | -0.561 to -0.178 | 0.0003 |
| SCML2 | -0.34 | -0.555 to -0.0718 | 0.0115 | -0.21 | -0.452 to 0.0672 | 0.1261 |
| SENP8 | -0.48 | -0.637 to -0.29 | <0.0001 | -0.38 | -0.554 to -0.168 | 0.0005 |
| SPATA12 | -0.52 | -0.665 to -0.334 | <0.0001 | -0.43 | -0.594 to -0.226 | <0.0001 |
| SPIRE1 | -0.50 | -0.654 to -0.316 | <0.0001 | -0.39 | -0.566 to -0.185 | 0.0003 |
| STK33 | -0.29 | -0.482 to -0.0632 | 0.0105 | -0.20 | -0.409 to 0.0283 | 0.0766 |
| TDRD10 | -0.47 | -0.631 to -0.279 | <0.0001 | -0.34 | -0.526 to -0.127 | 0.0018 |
| TKTL2 | -0.44 | -0.605 to -0.243 | <0.0001 | -0.36 | -0.538 to -0.147 | 0.001 |
| TMEM215 | -0.52 | -0.663 to -0.329 | <0.0001 | -0.40 | -0.574 to -0.195 | 0.0002 |
| TTLL2 | -0.53 | -0.679 to -0.351 | <0.0001 | -0.47 | -0.63 to -0.274 | <0.0001 |
| UBN2 | -0.44 | -0.602 to -0.237 | <0.0001 | -0.38 | -0.555 to -0.17 | 0.0005 |
| UBQLNL | -0.51 | -0.654 to -0.317 | <0.0001 | -0.38 | -0.554 to -0.168 | 0.0005 |
| USP6 | -0.50 | -0.653 to -0.311 | <0.0001 | -0.39 | -0.564 to -0.177 | 0.0004 |
| ZNF280B | -0.51 | -0.661 to -0.325 | <0.0001 | -0.43 | -0.595 to -0.224 | <0.0001 |
|  |  |  |  |  |  |  |
| MBNL3 | 0.45 | 0.239 to 0.62 | <0.0001 | 0.37 | 0.143 to 0.555 | 0.0014 |
| WNK3 | 0.41 | 0.185 to 0.592 | 0.0005 | 0.27 | 0.027 to 0.478 | 0.0255 |

- Spearman correlation analysis
- An Unpaired two-tailed t-test was used to calculate the p-value.
- Significant change in abundance level was considered with a p-value < 0.05
