# Supplementary figures and images for: MicroRNA-targeting in male infertility: Sperm microRNA-19a/b-3p and its spermatogenesis related transcripts content in men with oligoasthenozoospermia
Source: Front Cell Dev Biol. 2022 Sep 21;10:973849. doi: 10.3389/fcell.2022.973849 (PMC9533736; doi:10.3389/fcell.2022.973849)

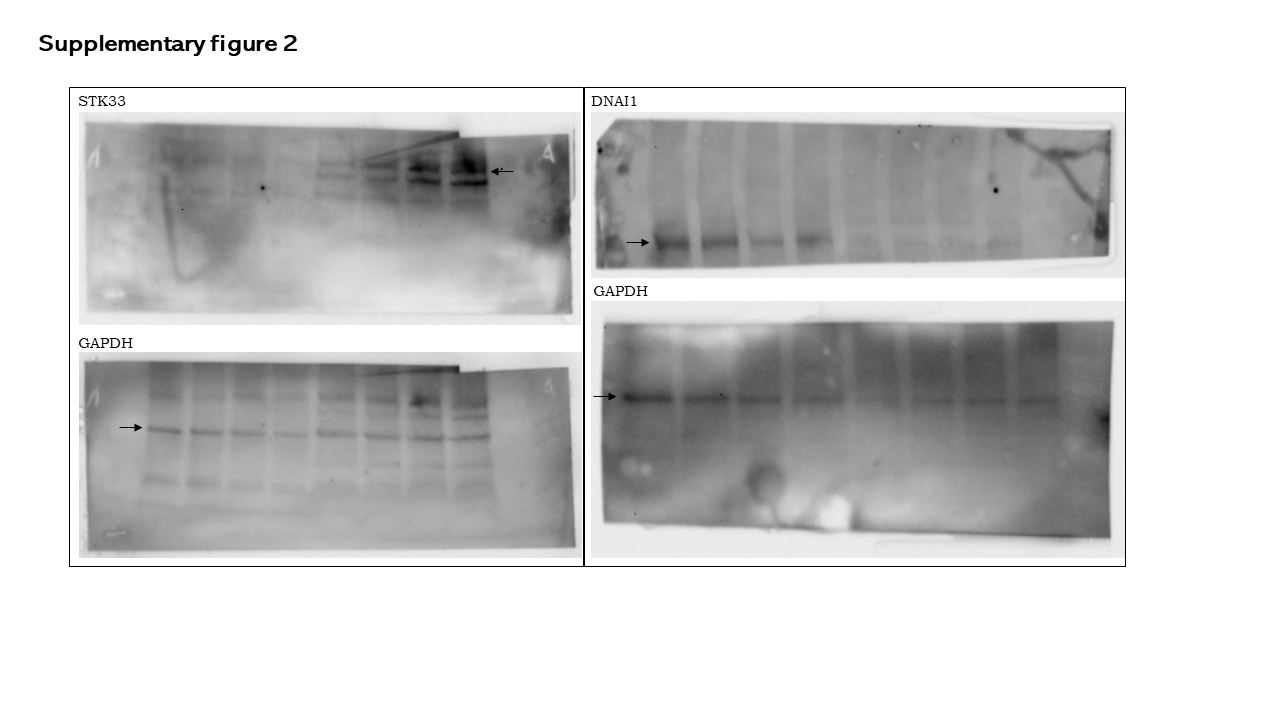

Supplement: Supplementary file 2 [file Image1.TIF]

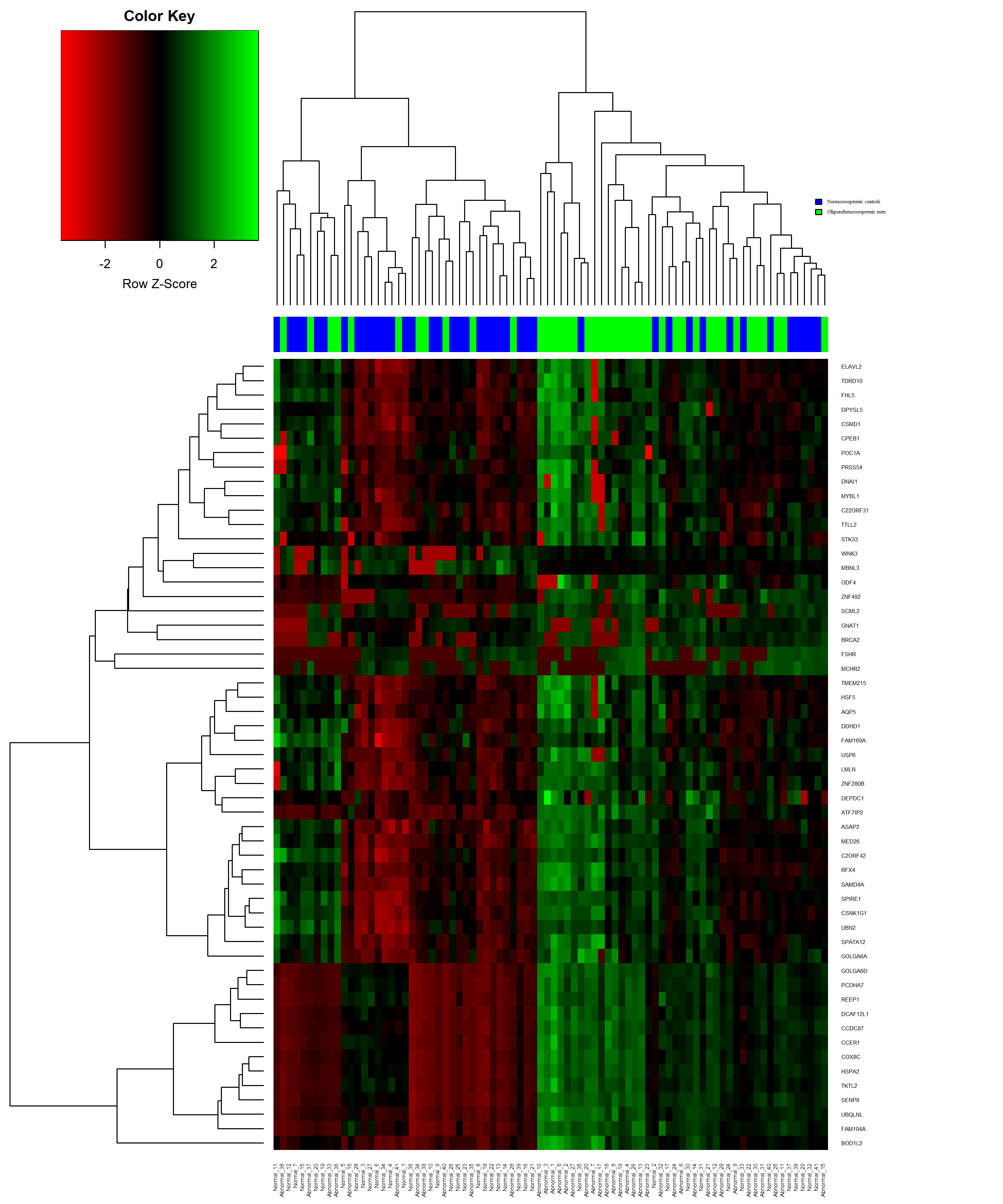

Supplement: Supplementary file 3 [file Image2.TIFF]
